# Differences in clinical and imaging characteristics between p16-positive non-smokers and p16-positive smokers or p16-negative patients in oropharyngeal carcinoma

**DOI:** 10.1038/s41598-021-82999-3

**Published:** 2021-02-08

**Authors:** Jean-Michel Trinh, Jacques Thomas, Julia Salleron, Philippe Henrot

**Affiliations:** Institut de Cancérologie de Lorraine, 6 Avenue de Bourgogne, 54519 Vandœuvre-lès-Nancy, France

**Keywords:** Head and neck cancer, Cancer imaging, Medical research

## Abstract

The eighth edition of the TNM classifies oropharyngeal squamous cell carcinomas (OSCCs) depending on p16 status. Some imaging features are reportedly associated more frequently with p16-positive (P16+) OSCC than p16-negative (p16−) OSCC. However, classical risk factors such as tobacco use were not specifically considered when assessing these imaging features. We aimed to evaluate whether P16+ OSCCs have different epidemiological, clinical, prognostic and imaging features depending on smoking status, and to compare P16+ and p16− groups. A retrospective study of data from 85 patients with P16+ OSCC (41 non-smokers, 44 smokers) and 36 with p16− OSCC from 2011 to 2020 was carried out, assessing epidemiological data, clinical aspects of the tumour and presence of adenopathy. Staging was assessed according to the seventh and eighth editions of the TNM. Compared with P16+ OSCC non-smokers, P16+ OSCC smokers had tumours that were less well-defined (36.6% vs 77.8%, p < 0.001), more ulcerated (85.4% vs 44.4%, p < 0.001) and more necrotic (53.7% vs 25%, p = 0.012). There was also less downstaging from N2 or N3 of the seventh edition of the TNM to N1 of the eighth edition for smokers than non-smokers (22.7% vs 43.9%, p = 0.042). Compared with p16− tumours, more P16+ tumours had well-defined contours (55.8% vs 22.2%, p = 0.001), were exophytic (89.6% vs 72.2%, p = 0.023), less necrotic (40.3% vs 80.6%, p < 0.001), less ulcerated (97.2% vs 66.2%, p = 0.006) and involved less muscle tissue (26.0% vs 47.2%, p = 0.027).P16+ OSCCs of smokers show clinical, imaging and prognostic differences with P16+ OSCCs of non-smokers.

## Introduction

The 8th edition of the TNM profoundly changes the classification of head and neck tumours, particularly for oropharyngeal squamous cell carcinomas (OSCCs). This latest edition provides two different classifications of OSCC depending on identification of the p16 protein by immunohistochemistry, which is a strong indicator of human papilloma virus (HPV)-induced cancer in the area of the oropharynx^1^.

Historically, risk factors for OSCC were considered to be primarily age, smoking and alcohol abuse^2,3^. Studies^4–6^ show that the incidence of OSCC in the United States is increasing, although tobacco and alcohol consumption are decreasing. This seems to be strongly linked to increasing rates of HPV infection in the oropharyngeal space, and the frequency of OSCC associated with HPV increased from 16.3% during the 1980s to 72.7% during the 2000s^6^. Elrefaey et al.^7^ have shown that HPV infection is now the primary cause of tonsillar cancer in Europe and North America.

Prognosis appears to be better in p16-positive (P16+) OSCC than in p16-negative (p16−) OSCC, as demonstrated by several studies^7–12^, even after adjustment for risk factors.

Some imaging features have been suggested to be associated more with P16+ OSCC than with p16− OSCC^13,14^. However, classical risk factors such as smoking status were not specifically taken into account in the assessment of these imaging features.

The aim of our study is to evaluate whether there are different epidemiological, clinical, prognostic and imaging features in P16+ OSCC that depend on smoking status, and to compare the tumour characteristics of P16+ and p16− OSCCs.

## Methods

Data from patients with head and neck cancer from January 2011 to January 2020 were retrieved from our institution database by using “oropharyngeal cancer”, “palatine tonsil”, “base of tongue” and “HPV16” as search terms. p16 status was determined by anatomopathological examination of a biopsy of the lesion or of the resected lesion after surgery. Exclusion criteria were no pre-treatment CT available and tumour in a location other than the oropharyngeal palatine tonsils or base of tongue.

**Figure 1:**
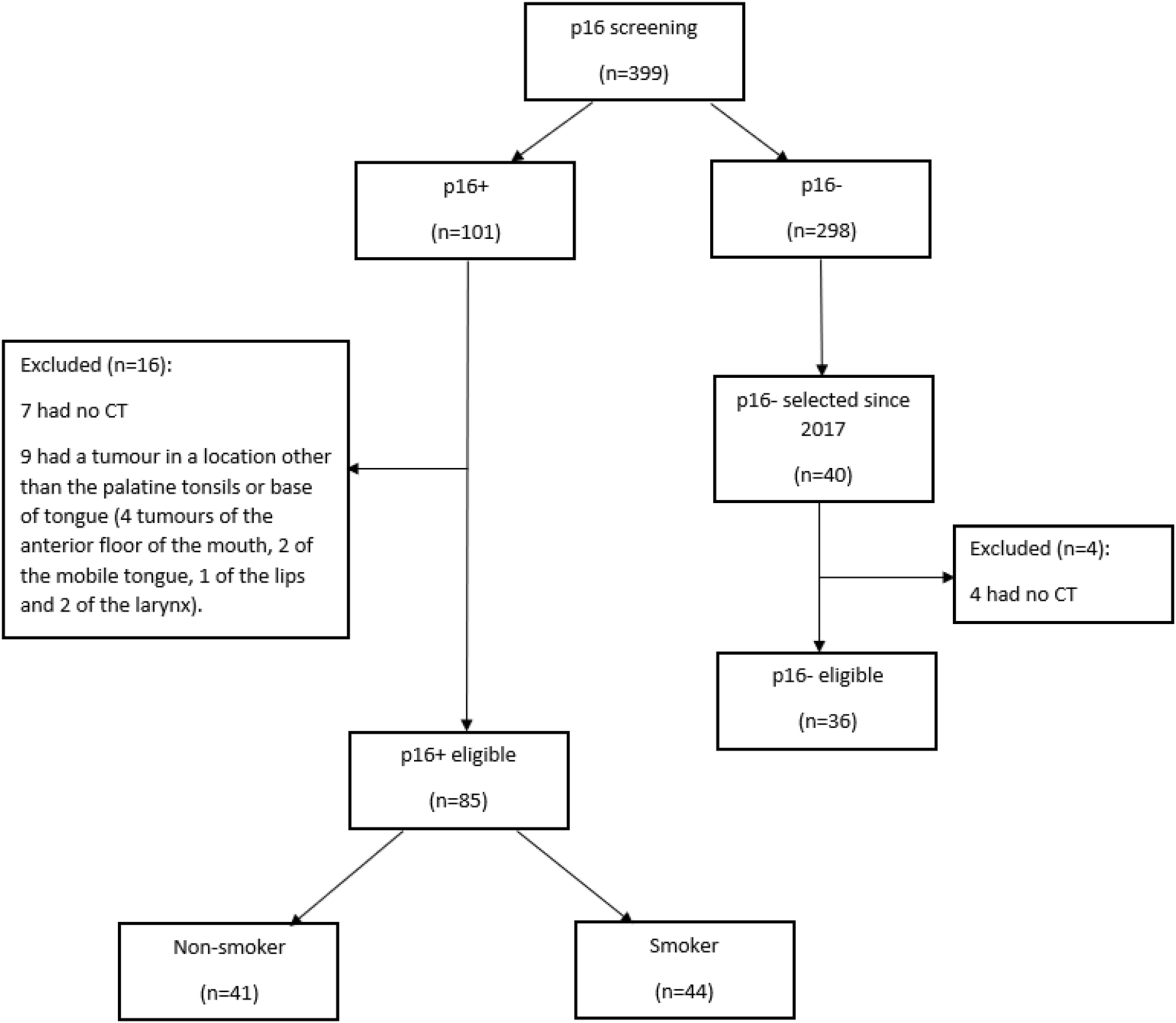
Flowchart of the patient selection process.

Of the 101 patients with P16+ OSCC, 85 fulfilled our inclusion criteria (Fig. 1) and two groups were considered: smokers and non-smokers (patients who had never smoked or had quit smoking for more than 20 years).

To obtain three groups of comparable size, we selected the first 40 consecutive patients with p16− status from January 2017 onwards (before this date, data are more often incomplete). Four patients were excluded because no pre-treatment CT images were available.

### Research of HPV infection status

Research of HPV was done on demand before 2017 and systematically from 2017 on patients who had an OSCC. p16 was detected by immunohistochemistry and sometimes complementary high-risk HPV PCR is performed to confirm unclear p16 status. p16 overexpression was considered as a surrogate to HPV infection^7^. As reported by Pannone et al.^15^, the p16 immunohistochemistry method proved a 100% sensitivity rate in OSCC confirmed HPV positive by PCR and/or In Situ Hybridation and a 93.5% specificity.

### Acquisition of images

CT with iodine contrast enhancement was used to acquire images from the base of the skull to the base of the cervicothoracic junction, followed by a thoracic CT. Dynamic maneuvers, such as opening of the mouth or opening of the mouth with extension of the tongue were occasionally performed to improve the image quality (in the case of metal artefacts) and the depiction of abnormalities^16,17^.

### Analysis of images

Images were displayed on a PACS workstation (Fujifilm Synapse v4.1.600, Fujifilm, USA) and assessed by a radiologist with 4 years’ clinical experience. CT and medical records were read from April 2019 to May 2020.

### Analysis of patient and tumour characteristics

The following data were collected from the medical files: sex, age at diagnosis of the cancer, tobacco consumption, pack-year, years since quitting smoking, alcohol habitus and p16 overexpression. Patients were considered drinkers or non-drinkers on the basis of clinicians’ reports.

Classification of the local extent (T) of the tumour was carried out according to both the 7th and 8th editions of the TNM by pooling together T1–T2, and T3–T4 stages. The primary site of the tumour was recorded (palatine tonsils, base of tongue), and subsequent extension towards oropharyngeal sublocations (tonsils, base of tongue, floor of the mouth, soft palate). Presence of metastases was also noted and reported according to the TNM.

**Figure 2:**
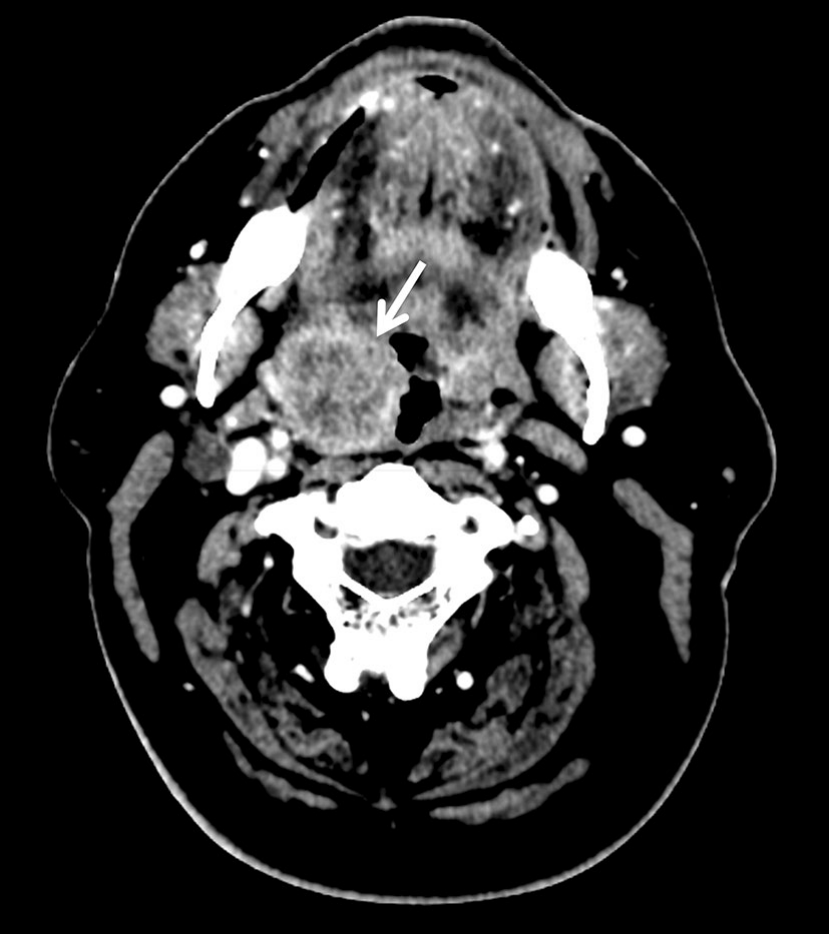
Axial CT slice showing well-defined contours of an exophytic P16+ tumour of the right palatine tonsil (arrow) in a 57-year-old male non-smoker.

**Figure 3:**
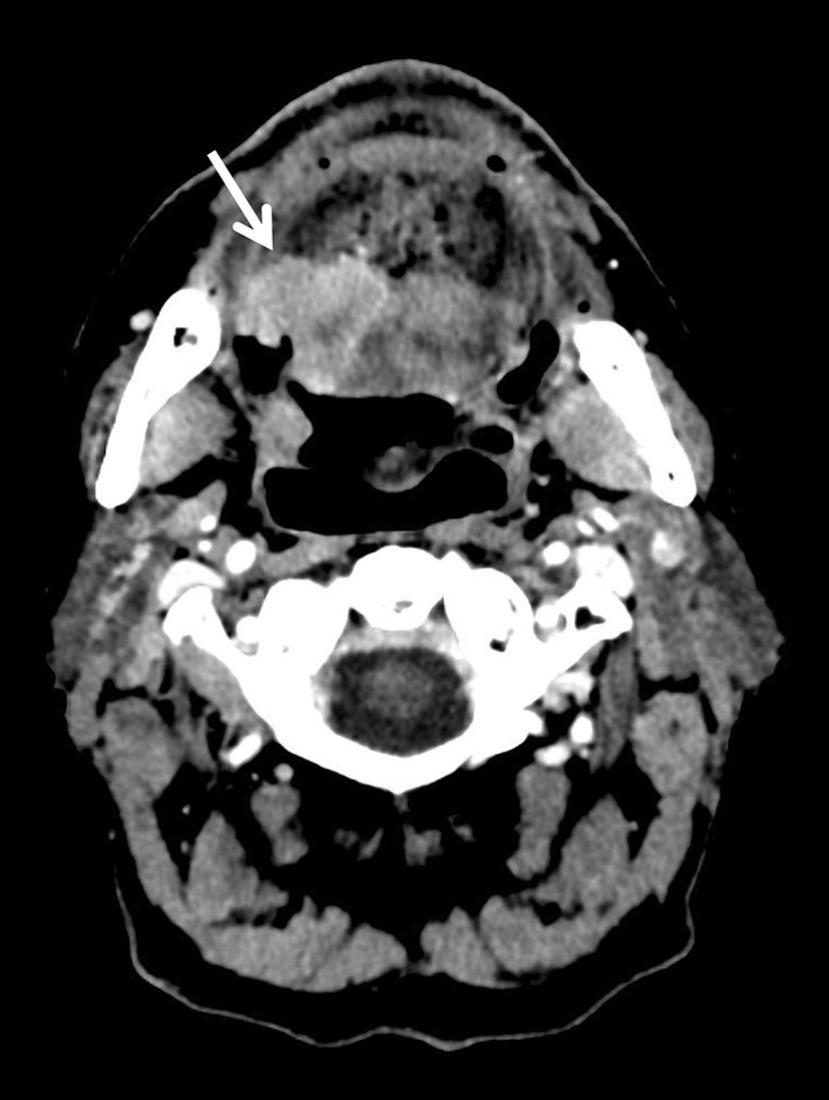
Axial CT slice showing a necrotic ulcerated (arrow) p16− tumour of the base of the tongue in a 59-year-old male former smoker.

**Figure 4:**
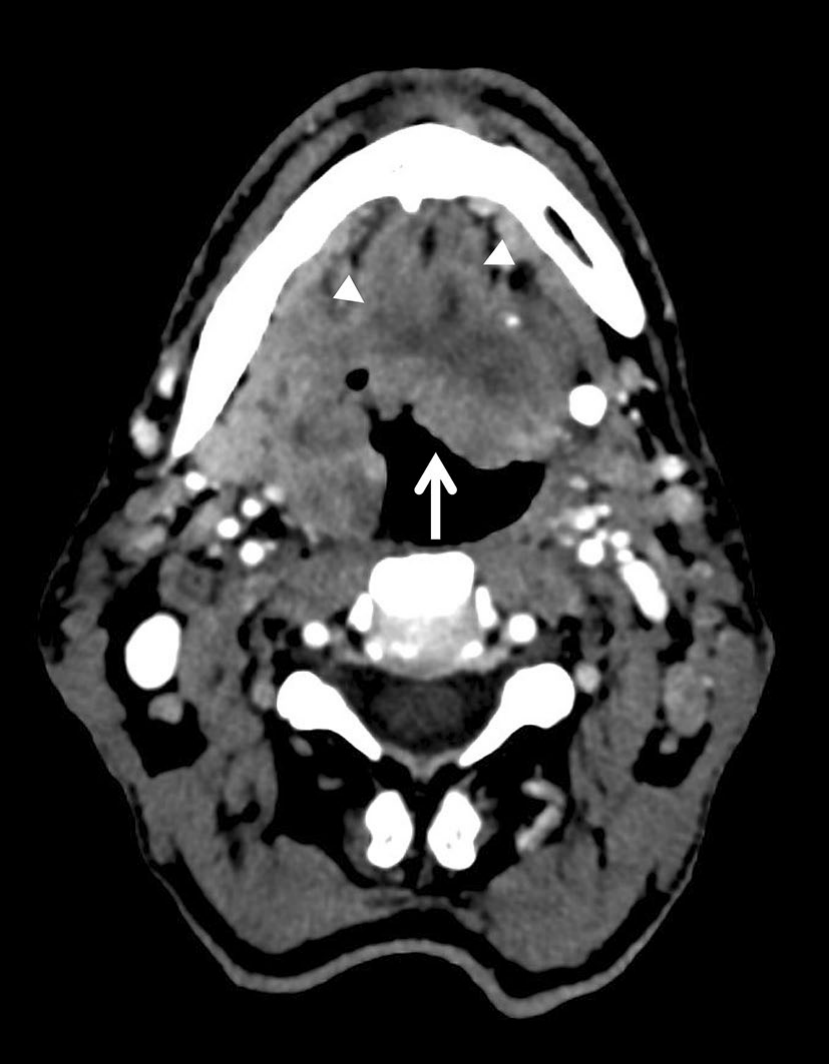
Axial CT slice showing ill-defined contours of a necrotic ulcerated p16− tumour (arrow) of the right palatine tonsil involving the base of the tongue and floor of mouth in a 53-year-old male smoker. Genioglossus muscles were also involved (arrowheads).

Morphological features of the tumour were also assessed: well-defined contour (Fig. 2), exophytic (Fig. 2), ulceration (Figs. 3, 4) and necrotic lesions (Figs. 3, 4). Extension to muscle was also noted (Fig. 4).

**Figure 5:**
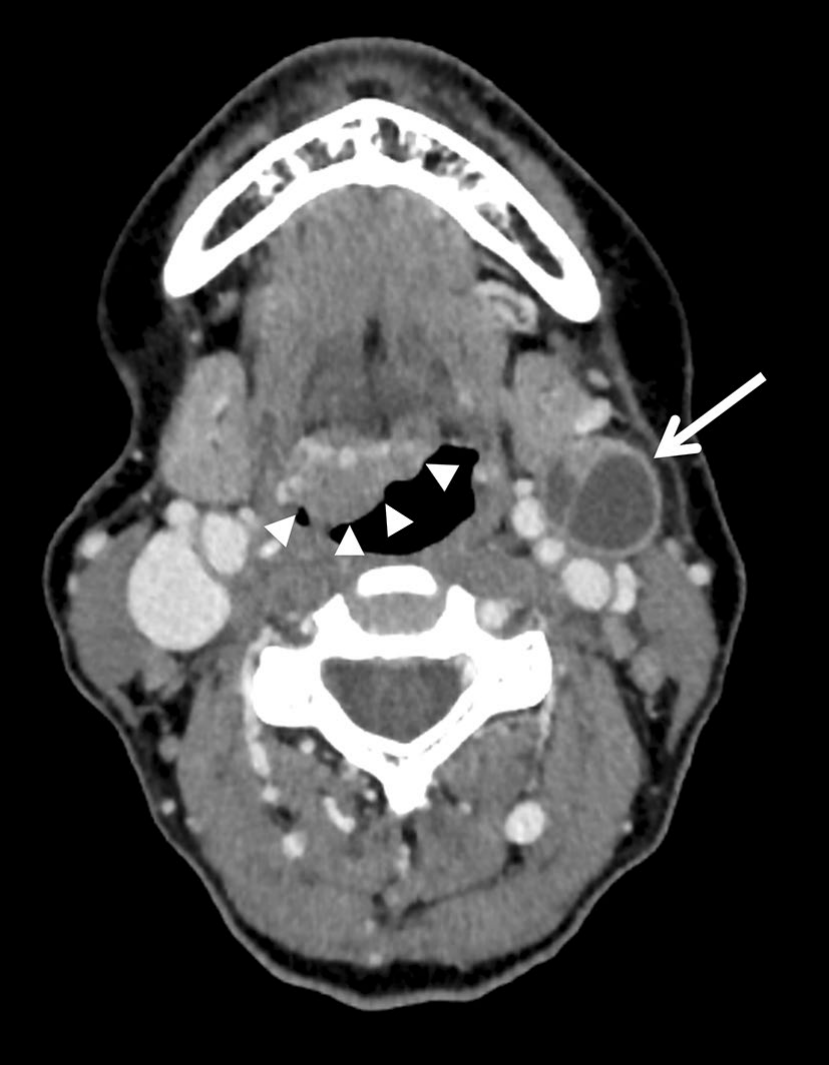
Axial CT slice showing a P16+ tumour of the base of tongue (arrowheads) and a typical cystic node without extra-nodal extension in the IIa left area (arrow) of a 53-year-old female with no history of tobacco or alcohol abuse.

Adenopathy was also assessed based on N of the TNM staging system (both 7th and 8th TNM editions); as N0, N1 or N2–N3 (N2 and N3 were grouped together). Morphological characteristics of adenopathy were also recorded: bilaterality, extra-nodal extension and cystic nodes (Fig. 5). Keratinizing histological type was also assessed.

### Statistical analysis

Quantitative parameters were the mean and standard deviation, and qualitative parameters were frequency and percentage.

Description of each parameter was assessed in P16+ and p16− group. Bivariate logistic regressions were performed to define parameters associated with P16+ status. Results were expressed as odds ratio and 95% confidence interval. Parameters with a p-value of less than 0.1 were introduced in a multivariate logistic regression with backward selection. The stability of the final model was investigated with Bootstrap resampling. The discriminant power of the final model was evaluated with the area under the curve (AUC) and was considered to be good if the AUC was greater than 0.8.

The same method was applied to compare data from P16+ smokers and non-smokers.

Statistical analysis was performed with SAS software, version 9.4 (SAS Institute Inc., Cary, NC, USA). The significance level was set at 0.05.

### Ethical approval

The study has been declared to the French National Commission on Information Technology and Liberties (CNIL) on April 12 2019 and has been registered as a CNIL compliance declaration (MR004-2203860) by the Data Protection Officer of the Cancer Institute of Lorraine French Region (Number 165). According to the CNIL MR004 compliance declaration, all patients are informed of the potential retrospective use of their clinical data for research purpose and of their right to refuse. Individual written consent was consequently waived in light of those elements, in compliance to CNIL MR004 declaration and the study was approved by the scientific and ethical committee of the Institut de Cancérologie de Lorraine, Nancy, France.

## Results

Among the 85 patients with P16+ OSCC, 44 (51.8%) were smokers. Median tobacco consumption was 34 pack-years in P16+ smokers and 40 pack-years in p16− patients. Sixteen patients among the 41 non-smokers with P16+ OSCC were former smokers (median time since quitting, 27.5 years). Characteristics of patients are presented in Table 1. Comparison of data from P16+ and p16− patients are presented in Table 2. Comparison of data from P16+ non-smokers and P16+ smokers are presented in Table 3.

**Table 1:** Characteristics of patients (n = 121).

Variables	Total n = 121
**P16+ **	85 (70.3)
P16+ non-smoker	41 (33.9)
P16+ smoker	44 (36.4)
p16−	36 (29.7)
Age (SD)	61.4 (9.9)
**Sex**	
Male	90 (74.4)
Female	31 (25.6)
Alcohol abuse	40 (33.3)
**Tumour (T) classification according to the 7th and 8th editions of the TNM**	
T1–T2	68 (56.2)
T3–T4	53 (43.8)
**Node (N) classification according to the 7th edition of the TNM**	
N0	25 (20.7)
N1	24 (19.8)
N2–N3	72 (59.5)
**Node (N) classification according to the 8th edition of the TNM**	
N0	25 (20.7)
N1	50 (41.3)
N2–N3	46 (38)
**Metastasis (M) according to the 7th and 8th editions of the TNM**	
M1	15 (12.4)
**Staging according to the 7th edition**	
Stage I–II	13 (10.7)
Stage III–IV	108 (89.3)
**Staging according to the 8th edition**	
Stage I–II	63 (52.1)
Stage III–IV	58 (47.9)
**Evolution of node staging from the 7th to 8th edition of the TNM (P16+ and p16− classification gathered)**	
N0 to N0	25 (20.7)
N1 to N1	22 (18.2)
N1 to N2 or N3	28 (23.1)
N2 or N3 to N1	44 (36.4)
N2 or N3 to N2 or N3	2 (1.7)
Well-defined contour^a^	51 (45.1)
Exophytic aspect of the tumour^a^	95 (84.1)
Ulceration^a^	86 (76.1)
Necrosis^a^	60 (53.1)
Muscle’s extension^a^	37 (32.7)
Bilateral adenopathy^b^	27 (28.1)
Extra-nodal extension^b^	37 (38.5)
Cystic adenopathy^b^	66 (68.8)
Keratinized histology at anatomopathological exam	33 (27.7)
**Tumour involvement (not exclusive)**	
Palatine tonsils	102 (84.3)
Base of tongue	61 (50.4)
Floor of mouth	32 (26.5)
**Primary lesion**	
Palatine tonsils	58 (47.9)
Base of tongue	20 (16.5)
Both	43 (35.5)

Variables are displayed as number (%) or mean (standard deviation).

^a^Data missing for 8 patients owing to no visible tumour (not visible clinically or on CT; pT1 on anatomopathological exam).

^b^Data missing for 25 patients owing to no visible adenopathy (N0).

**Table 2:** Comparison of data from P16+ and p16− patients (n = 121).

Variables	P16+ n = 85	p16− n = 36	OR and 95% CI (factors associated with P16+)	P test
Age (SD)	61.2 (9.7)	61.8 (10.5)	0.99 [0.96;1.03]	0.736
Alcohol abuse	18 (21.2)	22 (62.9)	0.16 [0.07;0.38]	< .001
**Tumour (T) classification according to the 7th and 8th editions of the TNM**				< 0.001
T1–T2	57 (67.1)	11 (30.6)	1	
T3–T4	28 (32.9)	25 (69.4)	0.22 [0.09;0.5]	
**Node (N) classification according to the 7th edition of the TNM**				0.141
N0	17 (20.0)	8 (22.2)	1	
N1	21 (24.7)	3 (8.3)	3.29 [0.76;14.37]	
N2–N3	47 (55.3)	25 (69.4)	0.89 [0.34;2.34]	
**Node (N) classification according to the 8th edition of the TNM**				< 0.001
N0	17 (20.0)	8 (22.2)	1	
N1	49 (57.6)	1 (2.8)	23.05 [2.68;198.0]	
N2–N3	19 (22.4)	27 (75)	0.33 [0.12;0.92]	
**Metastasis (M) according to the 7th and 8th editions of the TNM**				0.002
M1	5 (5.9)	10 (27.8)	0.16 [0.05 ;0.52]	
**Staging according to the 7th edition of the TNM**				0.932
Stage I–II	9 (10.6)	4 (11.1)	1	
Stage III–IV	76 (89.4)	32 (88.9)	1.06 [0.30;3.68]	
**Staging according to the 8th edition of the TNM**				< 0.001
Stage I–II	59 (69.4)	4 (11.1)	1	
Stage III–IV	26 (30.6)	32 (88.9)	0.06 [0.02;0.17]	
Well-defined contour^a^	43 (55.8)	8 (22.2)	4.43 [1.79;10.95]	0.001
Exophytic aspect of the tumour^a^	69 (89.6)	26 (72.2)	3.32 [1.18;9.32]	0.023
Ulceration^a^	51 (66.2)	35 (97.2)	0.06 [0.01;0.43]	0.006
Necrosis^a^	31 (40.3)	29 (80.6)	0.16 [0.06;0.42]	< .001
Muscle’s extension^a^	20 (26.0)	17 (47.2)	0.39 [0.17;0.90]	0.027
Bilateral adenopathy^b^	14 (20.6)	13 (46.4)	0.30 [0.12;0.77]	0.012
Extra-nodal extension^b^	26 (38.2)	11 (39.3)	0.96 [0.39;2.36]	0. 923
Cystic adenopathy^b^	51 (75.0)	15 (53.6)	2.60 [1.03;6.55]	0.043
Keratinized histology at anatomopathological exam	16 (19.3)	17 (47.2)	0.27 [0.11;0.63]	0.002
**Tumour involvement (not exclusive)**				
Palatine tonsils	74 (87.1)	28 (77.8)	1.92 [0.70;5.27]	0.204
Base of tongue	34 (40.0)	27 (75)	0.22 [0.09;0.53]	< 0.001
Floor of mouth	16 (18.8)	16 (44.4)	0.29 [0.12;0.68]	0.004
**Primary lesion**				0.002
Palatine tonsils	50 (58.8)	8 (22.2)	1	
Base of tongue	11 (12.9)	9 (25)	0.20 [0.06;0.62]	
Both	24 (28.3)	19 (52.8)	0.20 [0.08;0.53]	

Variables are displayed as number (%) or mean (standard deviation).

^a^Data missing for 8 patients owing to no visible tumour (not visible clinically or on CT; pT1 on anatomopathological exam).

^b^Data missing for 25 patients owing to no visible adenopathy (N0).

**Table 3:** Comparison of data from P16+ non-smokers and P16+ smokers (n = 85).

Variables	P16+ non- smoker n = 41	P16+ smokers n = 44	OR and 95% CI (factors associated with tobacco)	P test
Age (SD)	63.4 (9.7)	59.1 (9.4)	0.95 [0.91; 0.99]	0.004
Alcohol abuse	5 (12.2)	13 (29.6)	3.02 [0.97; 9.42]	0.057
**Tumour (T) classification according to the 7th and 8th edition**				0.487
T1–T2	29 (70.7)	28 (63.6)	1	
T3–T4	12 (29.3)	16 (36.4)	1.38 [0.56;3.43]	
**Node (N) classification according to the 7th edition**				0.031
N0	3 (7.3)	14 (31.8)	1	
N1	11 (26.8)	10 (22.7)	0.20 [0.04;0.88]	
N2–N3	27 (65.9)	20 (45.5)	0.16 [0.04;0.63]	
**Node (N) classification according to the 8th edition**				0.023
N0	3 (7.3)	14 (31.8)	1	
N1	29 (70.7)	20 (45.5)	0.15 [0.04;0.58]	
N2–N3	9 (22)	10 (22.7)	0.24 [0.05;1.11]	
**Metastasis (M) according to the 7th and 8th edition**				0.705
M1	2 (4.9)	3 (6.8)	1.43[0.23 ;9.00]	
**Staging according to the 7th edition**				0.117
Stage I–II	2 (4.9)	7 (15.9)	1	
Stage III–IV	39 (95.1)	37 (84.1)	0.27 [0.05;1.39]	
**Staging according to the 8th edition**				0.100
Stage I–II	32 (78.1)	27 (61.4)	1	
Stage III–IV	9 (22)	17 (38.6)	2.24 [0.86;5.83]	
**Evolution of node staging from 7 to 8th edition (P16+ and p16− classification gathered)**				0.042
N0 to N0	3 (7.3)	14 (31.8)	8.4 [1.94;36.42]	
N1 to N1	11 (26.8)	10 (22.7)	1.64 [0.52;5.19]	
N1 to N2 or N3	0 (0)	0 (0)	-	
N2 or N3 to N1	18 (43.9)	10 (22.7)	1	
N2 or N3 to N2 or N3	9 (22)	10 (22.7)	2.00 [0.61;6.55]	
Well-defined contour^a^	28 (77.8)	15 (36.6)	0.17 [0.06;0.45]	< 0.001
Exophytic aspect of the tumour^a^	34 (94.4)	35 (85.4)	0.34 [0.07;1.82]	0.209
Ulceration^a^	16 (44.4)	35 (85.4)	7.29 [2.46;21.63]	< .001
Necrosis^a^	9 (25)	22 (53.7)	3.47 [1.31;9.19]	0.012
Muscle’s extension^a^	9 (25)	11 (26.8)	1.10 [0.40;3.06]	0.855
Bilateral adenopathy^b^	6 (15.8)	8 (26.7)	1.94 [0.59;6.37]	0.275
Extra-nodal extension^b^	13 (34.2)	13 (43.3)	1.47 [0.55;3.94]	0.443
Cystic adenopathy^b^	29 (76.3)	22 (73.3)	0.85 [0.28;2.57]	0.778
Keratinized histology at anatomopathological exam	5 (12.8)	11 (25)	2.27 [0.71;7.23]	0.116
**Tumour involvement (not exclusive)**				
Palatine tonsils	35 (85.4)	39 (88.6)	1.34 [0.38;4.77]	0.654
Base of tongue	15 (36.6)	19 (43.2)	1.32 [0.55;3.15]	0.535
Floor of mouth	7 (17.1)	9 (20.5)	1.25 [0.42;3.73]	0.691
**Primary lesion**				0.693
Palatine tonsils	26 (63.4)	24 (54.6)	1	
Base of tongue	5 (12.2)	6 (13.6)	1.30 [0.35;4.82]	
Both	10 (24.4)	14 (31.8)	1.52 [0.57;4.05]	

Variables are displayed as number (%) or mean (standard deviation).

^a^Data are missing for 8 patients owing to no visible tumour (not visible clinically or on CT; pT1 on anatomopathological exam).

^b^Data missing for 17 patients owing to no visible adenopathy (N0).

Patients with P16+ OSCC had lower stage tumours, classified according to the 8th edition of the TNM, than those with p16− OSCC (p < 0.001). Five P16+ non-smokers and three P16+ smokers were T1 at clinical examination and pT1 on anatomopathological examination with no visible tumour on CT. Morphological characteristics of the tumour were not assessed in these cases.

Compared to patients in the p16− group, patients in the P16+ group had more tumours with well-defined contours (55.8% vs 22.2%, p = 0.001), and their tumours were more exophytic (89.6% vs 72.2%, p = 0.023), less necrotic (40.3% vs 80.6%, p < 0.001), less ulcerated (97.2% vs 66.2%, p = 0.006) and involved less muscle tissue (26% vs 47.2%, p = 0.027).

Bilateral adenopathy was more frequent in p16− patients (46.4% vs 20.6%, p = 0.012), whereas cystic adenopathy was more frequent in P16+ patients (75% vs 53.6%, p = 0.043). Metastases were also more frequent in p16− patients (27.8% vs 5.9%, p = 0.002).

Tumours of patients in the P16+ group were less frequently keratinized on anatomopathological examination (19.3% vs 47.2%, p = 0.002).

There was a significant relationship between the primary location of the tumour and HPV status (p = 0.002), with more primary tumours located in the palatine tonsils in P16+ OSCC. Involvement of the base of the tongue and floor of mouth was less frequent in P16+ OSCC (p < 0.001 and p = 0.004, respectively).

In multivariate analysis, three factors were significantly associated with P16+ patients: alcohol abuse was less associated with P16+ patients (OR 0.23, 95% CI [0.09;0.59]) as was necrosis (OR 0.12, 95% CI [0.04; 0.39]), whereas exophytic tumours were associated more with P16+ patients (OR 5.74, 95% CI [1.35; 24.52]). The AUC was equal to 0.817.

Compared with P16+ non-smokers, P16+ smokers had more tumours with N0 and N2 or N3 status (respectively 31.8% vs 7.3% and 22.7% vs 22%) and fewer with N1 status (45.5% vs 70.7%, p = 0.023). Their tumours were also less well-defined (36.6% vs 77.8%, p < 0.001), more ulcerated (85.4% vs 44.4%, p < 0.001) and more necrotic (53.7% vs 25%, p = 0.012). There was also less downstaging from N2 or N3 of the 7th edition to N1 of the 8th edition of the TNM in smokers than non-smokers (22.7% vs 43.9%, p = 0.042).

Other characteristics were not significantly different between the two groups. In particular, non-keratinizing histology was not significantly different between smokers and non-smokers with P16+ OSCC in our population.

Multivariate analysis of data from the P16+ patients shows that two factors are significantly associated with smoking: in P16+ smokers, there were fewer tumours with well-defined contours (OR 0.3, 95% CI [0.1; 0.95]) and more tumours with ulceration (OR 4.1, 95% CI [1.22; 13.75]) than in P16+ non-smokers. The AUC was equal to 0.77.

## Discussion

This study confirms previously reported differences in clinical, epidemiological and imaging features in P16+ OSCC and p16− OSCC that were distinctly identified in the 8th edition of the TNM classification. This study also sheds light on differences in P16+ OSCC in smokers and non-smokers that may be considered separately in future staging models.

### Epidemiological review

In our study, P16+ smokers developed tumours sooner than P16+ non-smokers. Alcohol abuse was not significantly different between the two groups. A synergistic association of the two risk factors in the cancerogenesis could explain the occurrence of cancer at a younger age in our population. We defined non-smokers as people who had never smoked or who had quit smoking more than 20 years, as described by Marron and al.^18^. This study has shown that the risk of oropharyngeal cancer after 20 years of cessation of tobacco smoking is equivalent to that of people who have never smoked.

### Imaging features and anatomopathological considerations

Our study is the first to our knowledge to take into consideration historical risk factors such as smoking status and the relatively recently identified risk factor p16 status, and to compare imaging features between P16+ non-smokers, P16+ smokers and p16− patients. Previous studies have shown some imaging features that can help radiologists to distinguish P16+ OSCC and p16− OSCC: P16+ OSCCs are more likely to have well-defined borders, to be exophytic and have cystic nodes. Conversely, p16− OSCCs are likely to feature ill-defined borders, ulceration and necrosis. No significant difference was found for extra-nodal extension in P16+ OSCCs and p16− OSCCs^13,14^.

### Imaging features of P16+ OSCCs and p16− OSCCs

Our study shares some similar findings with the previous studies^13,14^: P16+ OSCCs are more often well-defined, exophytic, and less ulcerated and necrotic than p16− OSCCs. P16+ OSCCs also show less aggressive radiological features such as muscle tissue involvement. Adenopathy is also often more cystic and less often bilateral in P16+ patients than in p16− patients. P16+ OSCCs were significantly less often keratinized on anatomopathological examination than p16− OSCCs in our study, as described in the literature^19,20^. In multivariate analysis, three factors differed significantly between P16+ patients and p16− patients: alcohol abuse and tumour necrosis were associated less with P16+ patients than p16− patients, whereas exophytic tumours were associated more with P16+ patients than p16− patients. The AUC was equal to 0.817 and these three parameters could distinguish well the two groups.

### Imaging features of OSCCs of P16+ non-smokers and P16+ smokers

Our study reveals some differences in P16+ OSCCs depending on tobacco abuse that have not been reported previously to our knowledge. P16+ smokers have significantly fewer tumours with well-defined contours and their tumours are more often ulcerated and necrotic. In multivariate analysis of data from all eligible P16+ patients, two factors were found to be significantly associated with smoking: tumours with well-defined contours were less frequent in P16+ smokers and tumours with ulceration were more frequent. The AUC was equal to 0.77 and these two factors could help to distinguish the two groups. Non-keratinizing histology was dominant but not significantly different in non-smoker P16+ OSCC patients of our population.

### Treatment and prognosis

P16+ OSCC is generally considered to respond best to radiotherapy and chemotherapy^21,22^. Several studies have proposed de-escalation of irradiation dose and chemotherapy to reduce induced toxicity and complications in P16+ OSCC^22^. In our study, p16− patients had more advanced tumours associated with a worse prognosis than P16+ patients, consistent with the literature^7–12^. According to classification with the 8th edition of the TNM, P16+ patients had better prognosis; significantly more p16− patients had stage III or IV tumours than P16+ patients in our study. Our study also shows that P16+ smokers tend to have higher tumour staging than P16+ non-smokers according to the 8^th^ TNM classification; there were significantly more P16+ smokers with stage III or IV tumours than P16+ non-smokers. Considering the link between staging and prognosis, prognosis could be worse in P16+ smokers than in P16+ non-smokers. If this observation is confirmed in larger populations, treatment choice may need to take into consideration the smoking status. As expected, there was significant downstaging of P16+ tumours from the 7th to the 8th TNM classification. As the T criteria were not modified, the downstaging is mainly based on the N criteria. N2 ipsilateral unique or multiple adenopathy in the 7th TNM classification is now N1 in the 8th edition. For example, a T1–T2N2b in the 7th edition became a T1–T2N1 in the 8th edition, and as a consequence the staging changes from IVa to I. However, this downstaging may in fact reflect the better prognosis of P16+ OSCC in the non-smoker group, as the P16+ smokers could have more aggressive lesions.

Our study also shows that there is a link between tobacco use and P16+ OSCC characteristics. There was also significantly more downstaging from N2 or N3 of the 7th edition to N1 of the 8th edition in the non-smoker group. This may mean that P16+ smokers have more aggressive lesion, either locally or by node involvement or metastasis.

### Limitations

Our study has several limitations. Tumour assessments were from CT images, which have lower contrast resolution than MRI but better spatial resolution. Our center used to perform CT first instead of MRI to cover the thoracic area in the same examination time. Extension to muscle is consequently more difficult to evaluate even if muscle stranding or fat adjacent to muscle stranding indicates likely muscle involvement. Categorization of patients as drinkers or non-drinkers was also based on clinical reports, but no reliable quantification was possible in our study. Moreover, alcohol abuse may be under-estimated by the patient. However, alcohol status was not significantly different in our population of P16+ patients, whatever their smoking status.

## Conclusions

OSCCs are currently stratified according to their p16 status in the 8th TNM classification, without taking into consideration historical risk factors such as tobacco and alcohol consumption. Our study shows that P16+ OSCCs associated with tobacco abuse may be more aggressive than P16+ OSCCs in non-smoker patients, although their estimated alcohol status was not significantly different. In future studies it will be interesting to assess the outcomes and overall life expectancy of the P16+ OSCCs depending on their smoking status. If large studies confirm our findings, historical risk factors could also be considered in the future elaboration of the next edition of the TNM classification.

## Abbreviations

OSCC: Oropharyngeal squamous cell carcinoma
P16+: P16 positive
P16−: P16 negative
HPV: Human papilloma virus
PCR: Polymerase chain reaction
AUC: Area under the curve
TNM: Tumour node metastasis
CT: Computed tomography
MRI: Magnetic resonance imaging
